# Enhanced pyruvate metabolism in plastids by overexpression of putative plastidial pyruvate transporter in *Phaeodactylum tricornutum*

**DOI:** 10.1186/s13068-020-01760-6

**Published:** 2020-07-10

**Authors:** Seungbeom Seo, Joon Kim, Jun-Woo Lee, Onyou Nam, Kwang Suk Chang, EonSeon Jin

**Affiliations:** Department of Life Science, Research Institute for Natural Sciences, Hanyang University, Seoul, 04763 Republic of Korea

**Keywords:** Biomass, Lipids, *Phaeodactylum tricornutum*, Plastidial pyruvate transporter, Metabolic engineering

## Abstract

**Background:**

The development of microalgal strains for enhanced biomass and biofuel production has received increased attention. Moreover, strain development via metabolic engineering for commercial production is being considered as the most efficient strategy. Pyruvate is an essential metabolite in the cells and plays an essential role in amino acid biosynthesis and de novo fatty acid biosynthesis in plastids. Although pyruvate can be a valuable target for metabolic engineering, its transporters have rarely been studied in microalgae. In this study, we aimed to identify the plastidial pyruvate transporter of *Phaeodactylum tricornutum* and utilize it for strain development.

**Results:**

We identified putative pyruvate transporter localized in the plastid membrane of *Phaeodactylum tricornutum*. Transformants overexpressing the pyruvate transporter were generated to increase the influx of pyruvate into plastids. Overexpression of a plastidial pyruvate transporter in *P. tricornutum* resulted in enhanced biomass (13.6% to 21.9%), lipid contents (11% to 30%), and growth (3.3% to 8.0%) compared to those of wild type during one-stage cultivation.

**Conclusion:**

To regulate the pyruvate influx and its metabolism in plastids, we generated transformants overexpressing the putative plastidial pyruvate transporter in *P. tricornutum*. They showed that its overexpression for compartmentalizing pyruvate in plastids could be an attractive strategy for the effective production of biomass and lipids with better growth, via enhanced pyruvate metabolism in plastids.

## Background

The demand for renewable and clean resources of energy is increasing worldwide [1, 2]. Apart from sustainable resources such as solar and wind energy, biofuels are also being considered as a practical resource as an alternative to petroleum products [3, 4]. Biofuels are derived from biomass and are classified into three generations. The first- and second-generations, which are derived from conventional resources, cause economic and political problems or impractical conversion cost to biofuels. To overcome these disadvantages, a third-generation of biomass has been developed using microorganisms [3]. Among the microorganisms, microalgae have emerged as an alternative biofuel feedstock [5]. Microalgae grow much more readily than territorial plants; produce valuable bioresources such as carbohydrates, proteins, pigments, and lipids; and sequester CO_2_. In addition, their cultivation does not lead to competition with food and economically important crops [6–8]. Renewable and sustainable biomass production from microalgae is a reasonable option for biofuel; however, there is a challenge for its commercial production, such as high production costs [9–11]. Increasing biomass production and lipid accumulation of microalgae could effectively reduce the costs. However, efficient biomass production needs optimal culture conditions, and lipid induction to enhance lipid contents in microalgae requires adverse culture conditions, which reduces growth and biomass production and takes lots of time [12, 13]. To overcome the limitation of microalgae in industrial applications, genetic modification and metabolic engineering of microalgae are necessary [14].

Among microalgae, the marine diatom *Phaeodactylum tricornutum* is considered to be a model organism for studying the production of bioresources [15, 16]. *P. tricornutum* is cultivated photoautotrophically and accumulates a high content of lipids [17]. Its whole-genome and transcriptome data are readily available [18, 19], and methods for its transformation and various molecular tools for its analysis have been developed [20–22]. Recently, genome editing tools have been established for *P. tricornutum* to modify specific target genes with transcription activator-like effector nucleases (TALENs) and CRISPR/Cas9 [23, 24]. Thus, it is convenient to choose target genes involved in metabolic pathways and regulate gene expression effectively in *P. tricornutum*. Therefore, *P. tricornutum* can be a suitable organism for metabolic engineering to enhance the production of lipids and biomass.

Several strategies have been developed to modify microalgae for producing next-generation biofuels from their biomass genetically. These strategies have been devised (1) to control the expression of enzymes or transcription factors associated with fatty acid biosynthetic pathways, (2) to increase the supply of reducing equivalents and precursors to pathways and lipid droplets, and (3) to block competitive pathways [25]. These strategies have been successful in achieving the production of sustainable and useful bioresources from *P. tricornutum*. For example, transformants overexpressing diacylglycerol acyltransferase (DGAT) had a higher lipid content compared to the wild-type cells [26]. Moreover, overexpression of glucose-6-phosphate dehydrogenase (G6PD) increased fatty acid synthesis and lipid accumulation [27], and knockout of UDP-glucose pyrophosphorylase with TALEN, exhibited a high TAG accumulation [23].

Another efficient strategy is to regulate the compartmentalization of metabolites. Pyruvate is a central intermediate associated with many metabolic pathways, including the TCA cycle, glycolysis [28, 29], biosynthesis of amino acids [30], terpenoids [31], and de novo fatty acids [28, 29]. However, pyruvate transporters that deliver pyruvate to mitochondria or plastids have been only identified in yeast, drosophila, mammalian cells, and plants [32–35]. Plastidial pyruvate transporters, especially in plants, are characterized by their function [35]. A novel gene, bile acid:sodium symporter family protein 2 (*BASS2*), was identified through analysis of differential transcriptome between C3 and C4 plants. BASS2 recombinant protein transported pyruvate to plastids in a sodium-dependent manner [35]. In *Arabidopsis thaliana*, seed-specific overexpression of BASS2 resulted in 10–37% more lipid content in seeds and the transformants had 24–43% more oil yield than the wild type. *A. thaliana* BASS2 overexpression was reported to increase the transporter of pyruvate to the plastid, where an increase in pyruvate levels increased fatty acid biosynthesis [36].

In microalgae, pyruvate transporters have been rarely studied. In this paper, we sought to investigate the effect of overexpressing a plastidial pyruvate transporter (pyruvate transporter-plastid type, PtPTP) in *P. tricornutum*. As stated above, overexpression of plastidial pyruvate transporter had a noticeable effect in plants, which implies that an enhanced influx of pyruvate to plastids resulted in increased lipid production [36]. As a similar strategy, we analyzed the effect of overexpressing endogenous putative pyruvate transporter in *P. tricornutum* and demonstrated the enhanced production of biomass and lipids, simultaneously.

## Results and discussion

### Identification of the pyruvate transporter-plastid type (*PtPTP*) gene in *P. tricornutum*

**Fig. 1:**
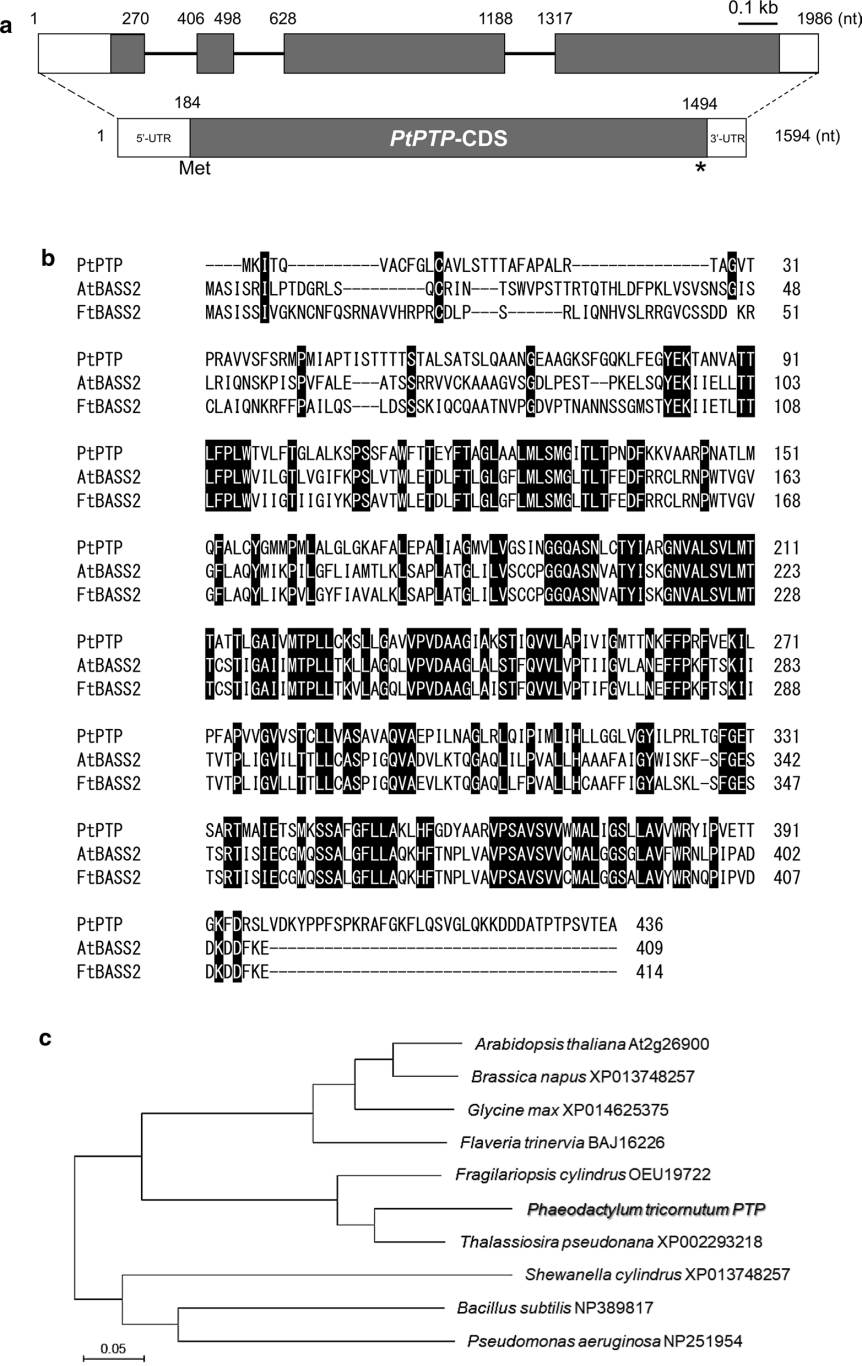
Characterization of *PtPTP* gene sequences in *P. tricornutum.***a** Map of genomic structure and transcript of *PtPTP*. Empty boxes are untranslated region (UTR), black-filled boxes are exons, and black lines are introns. **b** Alignment of PtPTP protein sequences with AtBASS2 (NP_850089) and FtBASS2 (BAJ16226). **c** Phylogenetic tree of *PtPTP* with related species

Based on previous studies on the plastidial pyruvate transporter in plants, we assessed the pyruvate transporter-plastid type gene in *P. tricornutum*. To identify the gene encoding the pyruvate transporter-plastid type in *P. tricornutum*, we first performed a sequence similarity search using BLAST analysis in the NCBI and JGI databases for *P. tricornutum* (https://mycocosm.jgi.doe.gov/Phatr2/Phatr2.home.html). The protein similarity test to the *Arabidopsis thaliana* plastidial pyruvate transporter revealed that *AtBASS2* (At2g26900), the candidate gene of PHATRDRAFT_3046 (GenBank XP_002179421), showed the highest similarity (49% protein identity and 65% protein similarity). However, this is only a partial gene sequence that lacks the start codon. We, therefore, cloned the full-length pyruvate transporter (*PtPTP*) sequence using 5′- and 3′-RACE analysis from the corresponding cDNA (Fig. 1a). In the genomic sequence of *P. tricornutum*, *PtPTP* has four exons, three introns, a 183-bp 5′-UTR, and a 99-bp 3′-UTR. The entire coding sequence (CDS) of *PtPTP* is 1311 bp. The complete amino acid sequence of PtPTP showed high similarity (62% and 61%, respectively) to that of other BASS2 proteins of the dicotyledonous C3 plant *A. thaliana* (At2g26900, NP850089) and C4 plant *Flaveria trinervia* (BAJ16226) (Fig. 1b). PTP-related protein sequences were derived from the NCBI database using BLAST with PtPTP, and a phylogenetic tree was constructed using the neighbor-joining method in the MEGA 10 software [37]. The phylogenetic tree indicated that *PtPTP* sequences have high similarity with those of the centric marine diatom *Thalassiosira pseudonana* and generate one cluster with the polar diatom *Fragilariopsis cylindrus*; they belong to different clades from those of higher plant groups and microorganisms (Fig. 1c). The characterization of the PtPTP sequence implied that the protein is conserved with high similarity and is closely related to those of other diatoms. With this putative transporter, we attempted to regulate the flux of pyruvate in metabolic pathways and engineer *P. tricornutum*. Its subcellular localization was further analyzed to clarify where the PtPTP functions as a transporter.

### Subcellular localization of PtPTP and generation of transformants overexpressing PtPTP

**Fig. 2:**
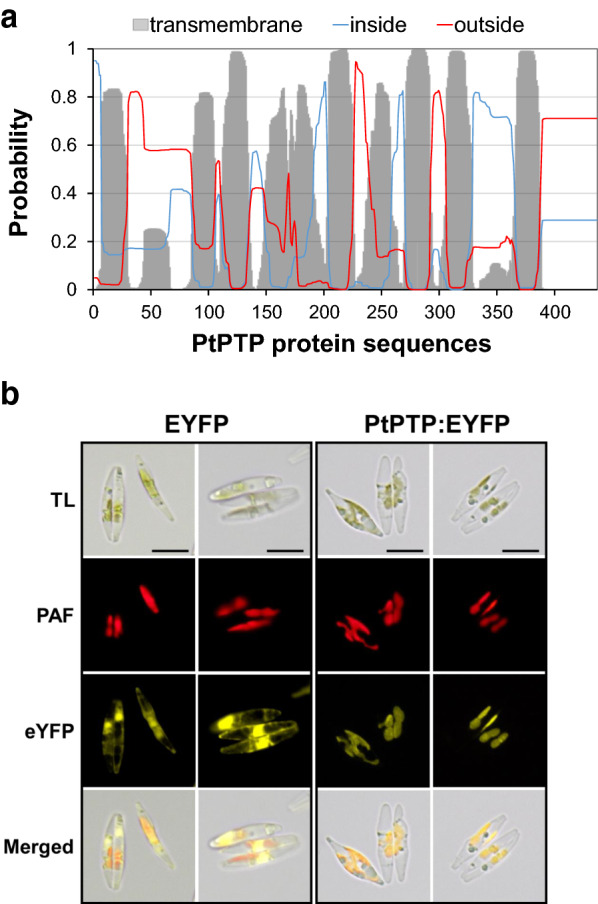
Subcellular localization of PtPTP in *P. tricornutum.***a** Membrane topology of PtPTP predicted using TMHMM Server v 2.0. Gray bar: transmembrane region, blue: region inside the membrane (cytoplasmic), red: region outside the membrane (exterior or luminal). **b** Observation of EYFP-expressing transformant and PtPTP::EYFP-expressing transformant through fluorescence microscopy. *TL* transmission light microscopic images, *PAF* plastid auto-fluorescence, *eYFP* enhanced yellow fluorescent protein. Merged: the above images overlaid. Scale bars 5 μm

To determine the function and structure of the PtPTP protein, we examined the transmembrane helices of the protein using TMHMM Server v. 2.0 (http://cbs.dtu.dk/services/TMHMM/). Examination revealed that PtPTP is a transmembrane protein that has 9 transmembrane helicase regions (Fig. 2a). Based on other prediction programs TargetP and ChloroP, the intracellular location of PtPTP protein was expected to be localized to chloroplasts and contain a signal peptide. TargetP indicated a higher probability (0.471) of the presence of a chloroplast transit peptide (cTP) than others [secretory pathway signal peptide [SP]: 0.446, mitochondria targeting peptide (mTP): 0.052] and predicted a potential presequence length of 63 amino acids. ChloroP also indicated that PtPTP had a 0.581 probability of the presence of a cTP with a length of 63 amino acids.

**Fig. 3:**
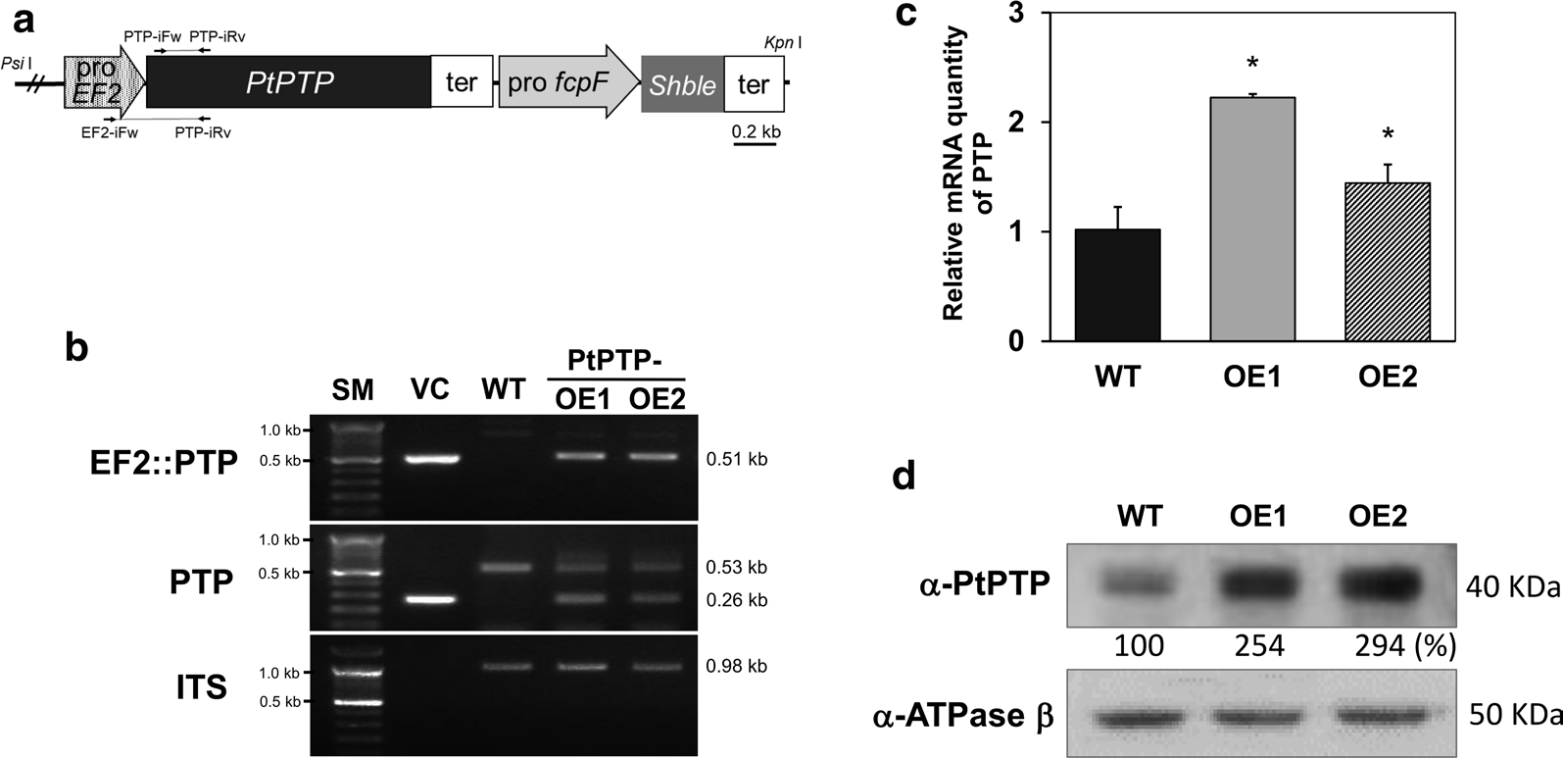
Generation of *PtPTP* overexpression strains and molecular analysis of *PtPTP* expression. **a** Map of *PtPTP* overexpression cassettes in pPhat-EF2-PtPTP. **b** Genomic PCR of the wild-type (WT) and *PtPTP*-overexpressing strains (PtPTP-OE1, OE2); Upper: PCR for EF2::PTP with primers of *EF2*-*iFw* and *PTP*-*iRv* (described in A); Middle: PCR for *PTP* with primers of *PTP*-*iFw* and *PTP*-*iRv* (described in A); Bottom: PCR for ITS (internal transcribed spacer) ITS, PCR for internal transcribed spacer (1.1 kb); SM, DNA size marker; VC, vector control; **c** Relative mRNA quantity of *PtPTP* in the wild-type (WT) and transgenic strains (OE1 and OE2). Error bars represent the mean value from three independent experiments. Statistically significant differences were determined by Student’s *t* test (***p < 0.001, **p < 0.01, *p < 0.05). **d** Immunoblotting with anti-PTP for analysis of overexpression of *PtPTP* in transgenic strains. Band intensities were quantified using Image J software, compared to the wild type

We previously constructed the pPhatT-EF2 expression vector harboring the elongation factor 2 (*EF2*) promoter from *P. tricornutum* for the generation of an overexpressing transgenic target gene [21]. The EF2 promoter triggers constitutive gene expression regardless of light/dark cycles [21]. To construct a transgenic transformant that causes continuous PTP gene overexpression using the *EF2* promoter, a transformation vector (pPhaT-EF2-PtPTP) capable of expressing an exogenous *PtPTP* coding sequence, PtPTP::EYFP (enhanced yellow fluorescence protein, EYFP, is fused to the C-terminus of PtPTP) or EYFP were constructed between the *EF2* promoter and *FcpA* terminator of the pPhaT-EF vector (Fig. 3a). To determine the subcellular localization of PtPTP, we generated transformants expressing EYFP and PtPTP::EYFP in *P. tricornutum*. PtPTP-EYFP fluorescence was detected in the chloroplast in the transgenic strains using fluorescence microscopy; plastid auto-fluorescence (PFA) indicated subcellular localization to the chloroplast. This corresponded precisely to the PtPTP-EYFP fluorescence. In contrast, in transformants expressing EYFP, the EYFP signal was dispersed in the cytoplasm (Fig. 2b). Based on the results of prediction and observation, the location and topology of PtPTP indicated it to be a plastid-localized transmembrane protein. Therefore, we might regulate the flux of pyruvate in plastids of *P. tricornutum* by manipulating the gene of putative pyruvate transporter localized in plastids.

For generation of PtPTP overexpression lines, zeocin-resistant colonies were screened using genomic PCR (Fig. 3b) to confirm the successful integration of the EF2::PtPTP expression cassette. For the positive control, the transformation vector (VC) was used as the PCR template. Amplification of genomic DNA by PCR using the primers *EF2*-*iFw* and *PTP*-*iRv*, flanking the *EF2* promoter region and *PtPTP*, revealed a 0.51-kb PCR product only in the transgenic strains (PtPTP-OE1 and OE2) (Fig. 3b). To identify the presence of both native endogenous *PtPTP* and the integrated EF2::PtPTP cassette in the genome, PCR using genomic DNA was performed with a specific primer set, *PTP*-*iFw* and *PTP*-*iRv* (Fig. 3a), which binds to the first and third exon regions containing two intron regions. The wild-type and transgenic strains presented large PCR bands (0.53 kb) containing 0.26 kb intron portions (Fig. 1a). However, the small PCR bands (0.26 kb) containing only the exon parts were shown only in transgenic strains harboring the exogenous coding sequence of *PtPTP*. When PCR was performed using primers of an internal transcribed spacer (ITS) gene as an internal control, the presence of the 0.98-kb PCR product was confirmed in both the wild-type strains and the transformants. These genomic DNA PCR results indicated that the exogenous *PtPTP* expression cassette of a transformation vector was successfully integrated into the genome of the transgenic strains.

Gene expression levels of the *PtPTP* gene were assessed by real-time qRT-PCR in the wild-type cells and two transformant cells cultured for 5 days after subculture at the transit point between the exponential to stationary phase. The relative transcript abundances of the transformants, PtPTP-OE1 and PtPTP-OE2, were about 2.2 and 1.4 times higher than that of the wild-type cells, respectively (Fig. 3c). This result demonstrated that two transgenic strains exhibited overexpressed levels of the *PtPTP* transcript compared to the wild type.

To confirm whether the increase in the expression level of the *PtPTP* transcript affects the amount of PtPTP protein, we assessed the relative amount of the PtPTP protein by immunoblot analysis using an antiserum against two specific synthetic peptides of PtPTP (Fig. 3d). The relative amount of the PtPTP protein in the two transformants to that in the wild type was examined using the anti-PtPTP antibody. The antibody reacted with a 40-kDa band corresponding to the predicted weight of PtPTP, with the deletion of 63 amino acids of the chloroplast transit peptide. The relative amounts of PtPTP protein increased in both PtPTP-OE1 (254%) and PtPTP-OE2 (294%), compared to that in the wild type (Fig. 3d). Finally, we successfully generated two transformants overexpressing the putative plastid-type pyruvate transporter. We expected that overexpressing plastidial pyruvate transporter encourages the influx of pyruvate into plastids and pyruvate metabolism in plastids. Thus, we further analyzed the phenotypes related to biomass and lipid production in the two transformants.

### The effects of overexpressing *PtPTP* on cell growth and their biomass production

**Fig. 4:**
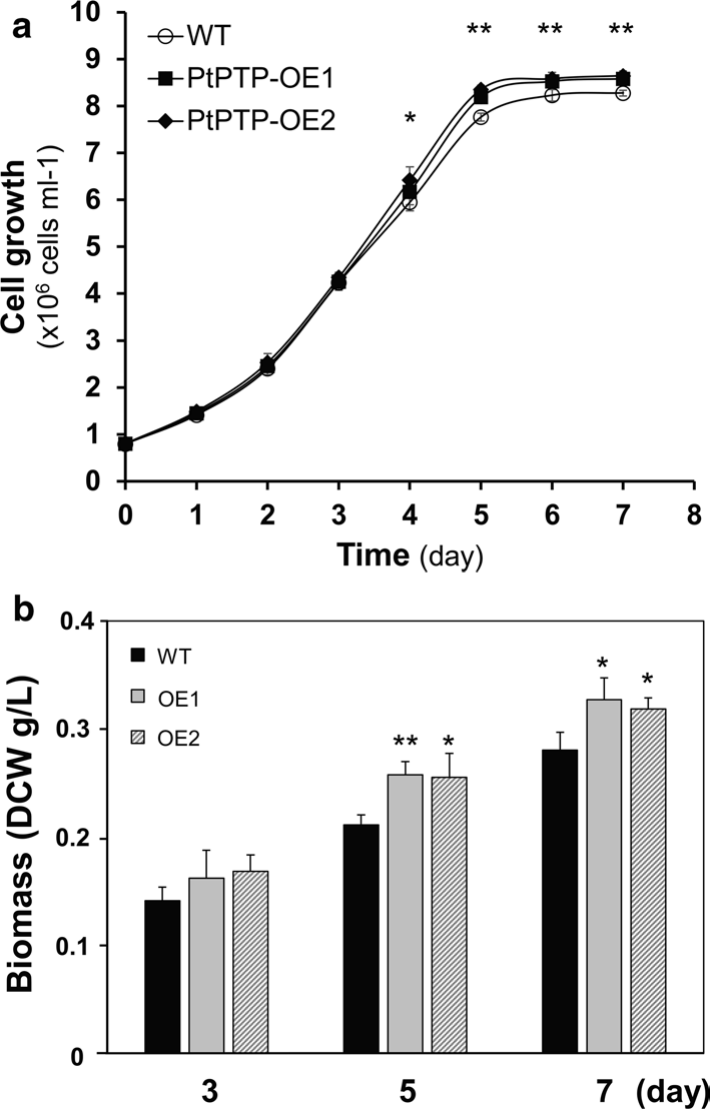
Growth and biomass production of the WT and transgenic strains. **a** Growth curves of the wild-type (WT) and *PtPTP* overexpression strains (PtPTP-OE1, PtPTP-OE2). Each curve shows the mean ± SD from three independent replicates. **b** Biomass production measured by calculating dry cell weight at days 3, 5, and 7 days of subculture. Error bars represent mean values from three independent experiments. Statistically significant differences were determined by Student’s *t* test (**p < 0.01, *p < 0.05)

The effects of overexpressing *PtPTP* in *P. tricornutum* were investigated by the analysis of cell growth and biomass production (Fig. 4). After inoculation with wild-type and transgenic strains at the same cell density of ~ 0.8 × 10^6^ cells/mL (OD_750_ ~ 0.05), we measured cell growth as a function of cell density. After subculture, their growth pattern showed exponential growth up to 5 days of subculture. Subsequently, the cells entered the stationary phase. After 3 days of subculture, the transgenic strains began to show a difference from the wild type in cell growth. In stationary phase, the number of wild-type cells reached 8.28 ± 0.06 × 10^6^ cells/mL and those of transgenic PtPTP-OE1 and PtPTP-OE2 were 8.58 ± 0.11 and 8.64 ± 0.10 × 10^6^ cells/mL, respectively. Independently repeated experiments (n = 3) of cell density showed the significantly increased cell density of PtPTP-OE1 from 5.5% (5 days, *p *< 0.01) to 3.3% (7 days, *p *< 0.01) and PtPTP-OE2 from 8.0% (4 days, *p *< 0.05) to 4.3% (7 days, *p *< 0.01) compared to that of the wild type (Fig. 4a).

The effects of *PtPTP* overexpression on biomass production were analyzed. While culturing cells in the flask, the biomass was estimated based on dry cell weight per culture volume in the exponential (3 days after subculture), the declining (5 days after subculture), and the late stationary growth phase (7 days after subculture) (Fig. 4b). Compared to the wild-type strain, transgenic PtPTP-OE1 and PtPTP-OE2 produced significantly more biomass at 5 and 7 days after inoculation. In the declining growth phase (5 days after inoculation), PtPTP-OE1 and PtPTP-OE2 produced 21.9 ± 0.01% and 20.8 ± 0.03% more biomass, respectively. At 7 days of the stationary phase, PtPTP-OE1 exhibited a 16.6 ± 0.04% increase in biomass and PtPTP-OE2 exhibited a 13.6 ± 0.03% increase in biomass. As a result, PtPTP-overexpressing strains showed higher cell density and more biomass production compared to that of their wild-type counterparts. Therefore, these engineered microalgae could serve as strains for efficient biomass production.

Comparing photosynthetic productivity and efficient quantum yield of photosystem II, Y(II), between transformants and wild type, the results of transformants were similar to those of the wild-type cells. This datum indicated that overexpression of pyruvate transporter seems not to affect photosynthetic activity (Additional file 1: Fig. S1). Hence, we predicted that enhanced biomass production is more a result of compartmentation of pyruvate and activated biosynthetic pathways in plastids.

### The effects of overexpressing *PtPTP* on lipid contents and fatty acids’ composition

**Fig. 5:**
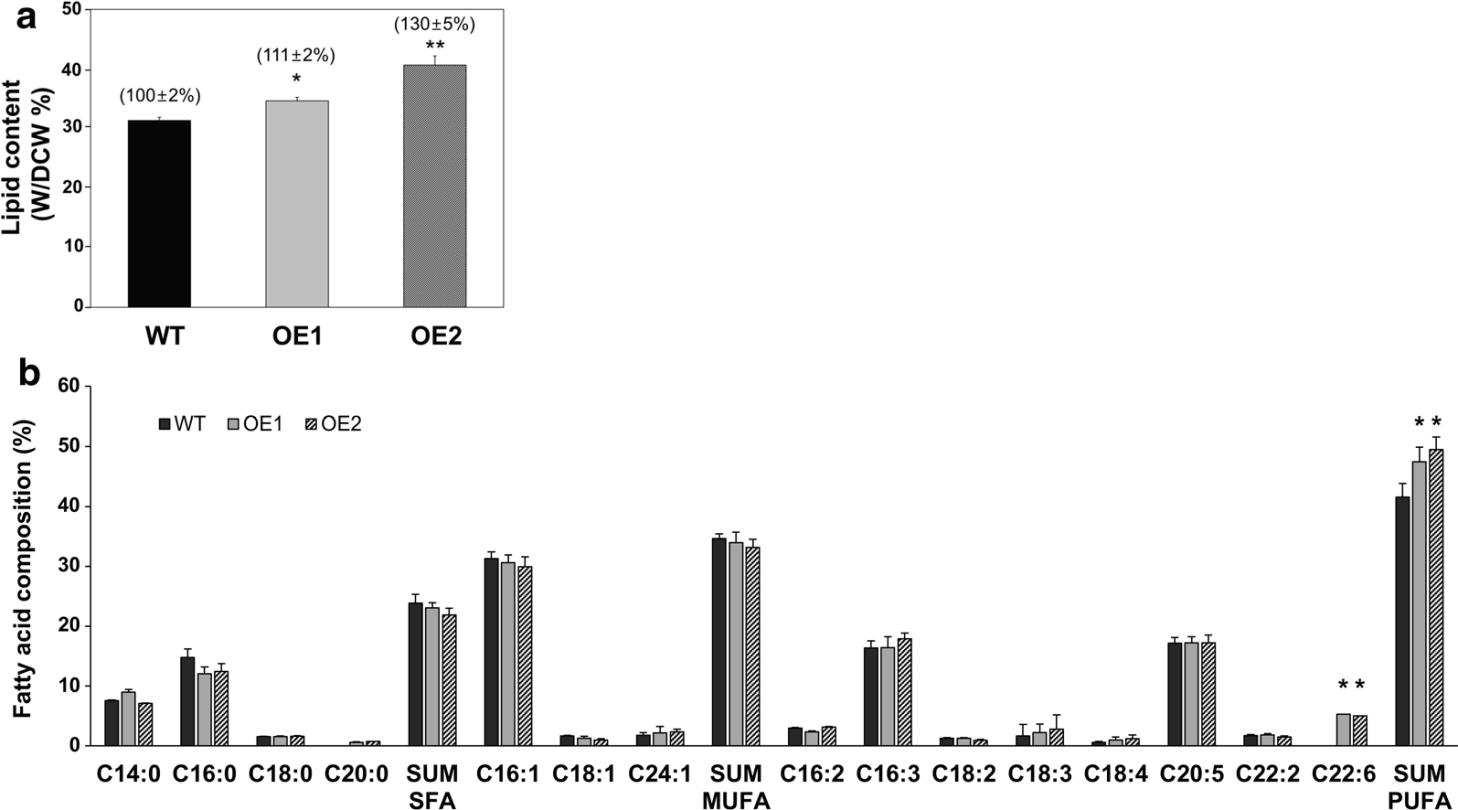
Lipid contents and fatty acid composition in the WT and transgenic strains. **a** Lipid contents were estimated at 7 days after subculture. The contents were calculated based on the dry cell weight. **b** Comparison of fatty acid composition in the WT and transgenic strains at 7 days after subculture. Error bars represent mean values from three independent experiments. Statistically significant differences were determined by Student’s *t* test (**p < 0.01, *p < 0.05)

The total lipid content per dry cell weight showed considerable accumulation at 7 days of the stationary phase as determined by the weighting method. PtPTP-OE1 and PtPTP-OE2 contained 111 ± 2% and 130 ± 5% of total lipids, respectively, compared to the wild type (Fig. 5a). Furthermore, FAME analysis using GC-MS revealed fatty acid composition in the wild-type and transgenic strains (Fig. 5b). A broad distribution of the fatty acids in the wild-type cells is similar to that of *PtPTP*-overexpressing transformants; however, there was a significant difference between the sum of polyunsaturated fatty acids (SUM PUFA) in each transformant. PtPTP-OE1 and PtPTP-OE2 (47.44% and 49.51%, respectively) had a higher PUFA content compared to the wild-type organism (43.39%). The high polyunsaturated fatty acid content in microalgae provides an advantage for the utilization of microalgal biomass for nutraceutical applications, pharmaceutical applications, and biodiesel production [38, 39].

### Transcriptional expression of genes related to pyruvate metabolism in plastids

**Fig. 6:**
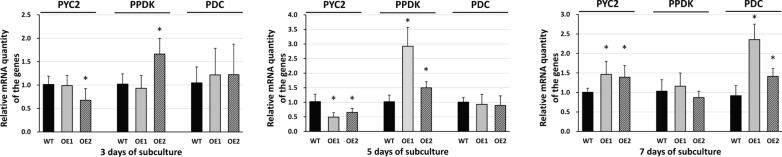
Quantitative real-time PCR for the genes related to pyruvate metabolism in plastids. Pyruvate dehydrogenase complex (PDC), pyruvate carboxylase 2 (PYC2) and pyruvate, phosphate dikinase (PPDK) transcript levels in WT and transgenic strains. Their gene expression levels at 3, 5, and 7 days of subculture were analyzed by qRT-PCR. The expression levels were normalized based on their TATA-box binding protein (TBP) expression (internal reference). Error bars represent mean values from three independent experiments. Statistically significant differences were determined by Student’s *t* test (*p < 0.05)

Based on the intracellular distribution of metabolic enzymes in *P. tricornutum*, several enzymes that utilize pyruvate as a substrate participate in pyruvate metabolism in plastids, such as pyruvate carboxylase 2 (PYC2), pyruvate–phosphate dikinase (PPDK) and pyruvate dehydrogenase complex (PDC) [40, 41]. PYC2 and PPDK could be involved in amino acids and lipids’ biosynthesis, producing oxaloacetate (OAA) and phosphoenolpyruvate (PEP), respectively [40, 42, 43]. PDC is an essential enzyme for de novo fatty acid biosynthesis in plastids [44]. PDC converts pyruvate to acetyl-CoA, generating NADH and CO_2_, and acetyl-CoA is a significant substrate for de novo fatty acid biosynthesis. By comparing the expression of their genes between wild type and transformants, we tried to estimate the effects of overexpressing putative pyruvate transporter on its metabolism in plastids. Using quantitative real-time PCR, we compared the expression level of PYC2 (GenBank XM_002183870), PPDK (GenBank XM_002182336), and PDC (pyruvate dehydrogenase complex; subunit E1, GenBank XM_002180298) between wild type and transformants at 3, 5, and 7 days after subculture (Fig. 6). At the 3 days after subculture, relative transcription levels of PDC did not show any difference between WT and transformants. PPDK transcript level in PtPTP-OE2 was up-regulated (1.63-fold), and its PYC2 transcript level was down-regulated (− 1.33-fold). At the 5 days after subculture, PDC transcript levels still did not show significant difference in transcription levels between WT and transformants (Fig. 6, 5 days of subculture). Transcription levels of PYC2 were down-regulated in PtPTP-OE1 (− 1.49-fold) and PtPTP-OE2 (− 1.66-fold), and those of PPDK were up-regulated in PtPTP-OE1 (2.93-fold) and PtPTP-OE2 (1.50-fold) (Fig. 6, 5 days of subculture). In the stationary phase (7 days after subculture), transcriptional expression levels of PDC were up-regulated in PtPTP-OE1 (2.54-fold) and PtPTP-OE2 (1.54-fold) (Fig. 6, 7 days of subculture). Transcriptional expression levels of PPDK in WT are similar with those in transformants, and PYC2 transcript levels are up-regulated in transformants compared to WT (PtPTP-OE1 1.46-fold, -OE2 1.39-fold) (Fig. 6, 7 days of subculture). As a result, increased biomass production in transformants might come from activated PPDK-related amino acids and lipid biosynthesis [30, 42], instead of PYC2-related biosynthesis until stationary phase. At the stationary phase, enhanced biomass and lipid production in transformants could be explained with up-regulated PDC in de novo fatty acid biosynthesis, and PYC-related biosynthesis might also contribute their increased biomass production in stationary phase. Enhanced biomass production and fatty acid biosynthesis in *PtPTP*-overexpressing strains seem to be related with these transcriptional expression patterns.

Based on these results, transformants showed increased productivity and the effects of several genes involved in pyruvate metabolism by overexpression of putative pyruvate transporter in plastids. Finally, we determined that engineering to regulate the influx of pyruvate into plastids by overexpression of *PtPTP* could enhance their biomass and lipid production, making this an attractive strategy to engineer microalgae.

## Conclusions

Overexpression of pyruvate transporter in *P. tricornutum* was studied for boosting the influx of pyruvate to plastids. The putative *PtPTP* localized in the plastid membrane was identified in *P. tricornutum*, based on the characterization of the sequences and analysis of its protein localization. Transgenic strains that constitutively overexpress *PtPTP* showed higher lipid contents and significantly better growth and biomass production than the wild type, enhancing pyruvate metabolism in plastids. These results demonstrate that compartmentation of pyruvate in plastids is an attractive strategy for metabolic engineering of *P. tricornutum* for biomass and biofuel production.

## Methods

### Strains and culture conditions

Axenic cultures of *P. tricornutum* Bohlin (CCMP632) were purchased from the National Center for Marine Algae and Microbiota (NCMA) at the Bigelow Laboratory for Ocean Sciences (East Boothbay, ME, USA). The cells were cultivated on a shaking incubator (130 rpm) in f/2 medium containing silicate (+ Si) (ASW), buffered with 40 mM Tris–HCl at pH 7.2; the cultures were maintained at 20–22 °C; under a 12:12, light:dark cycle. The light was provided by fluorescent lamps (~ 50 µmol m^−2^ s^−1^). As supplying inorganic carbon source to the cells is advantageous for cultivation, 10 mM bicarbonate was added into the culture medium.

### Construction of transformation vector

Plasmid PhaT-EF2-PtPTP was constructed from the previously designed plasmid pPhaT-EF2-Luc [21]. To delete the fragment encoding luciferase, pPhaT-EF2-Luc was digested with XbaI and SpeI. The full-length cDNA of PtPTP was amplified with specific primers (forward (Fw), 5′-TCTAGACCGTTGCAATACATCCCCCA-3′, the XbaI site is underlined; reverse (Rv), 5′-ACTAGTCGCTTCCGTCACCGAGGGTGTC-3′, the SpeI site is underlined) and cloned into pPhaT-EF2-Luc whose luciferase gene was deleted. For tagging PtPTP with enhanced yellow fluorescence protein (EYFP), the *eyfp* gene was amplified with the following primer set: Fw 5′-TCTAGATGAGCAAGGGCGAGGAGCTG-3′; the XbaI site is underlined and Rv 5′-ACTAGTTTACTTGTACAGCTCGTCCAT-3′; the SpeI site is underlined. The *eyfp* fragment was digested with XbaI and SpeI and cloned into the pPhat-EF2-PtPTP digested with SpeI, to generate pPhat-EF2-PtPTP:EYFP.

### Transformation and screening of transformed cells

M17 (1.1 μm in diameter) tungsten particles were coated with the transformation vector; 5 × 10^7^*P. tricornutum* cells were spotted on the center of f/2-Si 1.2% agar plates. Particle bombardment was carried out using a Biolistic Particle Delivery System PDS-1000/He (Bio-Rad Laboratories, CA, USA) fitted with 1550 psi rupture discs, as suggested by the manufacturer. The gene transfer protocol was adapted from that described by Falciatore et al. [45]. Bombardmented cells were spread onto an f/2 medium agar plate containing 100 μg/mL Zeocin, and resistant colonies appeared after 3–4 weeks. Colonies were cultured on fresh agar plates and screened by colony polymerase chain reaction (PCR), which was performed as follows. The seeded colonies from agar plates were suspended in distilled water and used as a template for PCR with rTaq 5 × PCR Master Mix (ELPIS-Biotech, Daejeon, Korea). Primer sets used for PCR of exogenous EF2::PTP and PTP are shown in Additional file 1: Table S1.

### Quantitative real-time PCR and immunoblotting

Quantitative real-time PCR (qRT-PCR) was carried out, as described by Seo et al. [21]. Total RNAs were extracted for the qRT-PCR analysis of the relative expression levels of *PtPTP* in the WT and transgenic strains. All values are shown as the mean of two technical replicates for each independently prepared biological sample (*n *= 3) with standard deviation. The *PtPTP* expression levels were normalized using the expression of the gene encoding their TATA-box binding protein (TBP) as an internal reference. Primer sets used for qRT-PCR are shown in Additional file 1: Table S1.

For immunoblotting, cells were harvested by centrifugation at 2000 *g* for 15 min. Pellets were suspended in extraction buffer (10 mM Tris–HCl, 1 mM EDTA, 0.2% SDS, and 1× protease inhibitor cocktail (Thermo Scientific, IL, USA). The cells were disrupted thoroughly by sonication (4 times, 30 s each). Extracted total protein was quantified using the Pierce BCA protein assay kit (Thermo Scientific, IL, USA) and loaded (20 μg per lane) onto a 10% SDS-PAGE gel for separation. Separated proteins were electro-transferred to a PVDF membrane for immunoblotting. The primary antibody specific to a mixture of two synthetic PtPTP peptides was prepared by Abfrontier (Abfrontier, Seoul, Korea). The sequences of two PtPTP peptides are H_2_N-SLQAANGEAAGKSFGQKLFEG-COOH and H_2_N-PVETTGKFDRSLVDKYPPFS-COOH. The antibody against PtPTP was used at a dilution of 1:5000. Anti-ATP-β was used as a loading control. The secondary antibody was used at a dilution of 1:20,000. Band intensities were quantified using the Image J software and expressed as relative intensity.

### Subcellular localization of PtPTP in *P. tricornutum*

To determine the subcellular localization of PtPTP in *P. tricornutum*, PtPTP:EYFP-expressing transformants and EYFP-expressing transformants were observed under a Nikon ECLIPSE Ni fluorescence microscope (Nikon, Japan). The EYFP signal was detected with an excitation filter (Ex) with a wavelength of 490–500 nm, a dichroic mirror (DM) with a wavelength of 510 nm, and an absorption filter (BA) with a wavelength of 520–560 nm. The auto-fluorescence signal was detected using an Ex with a wavelength of 540–580 nm, DM with a wavelength of 595 nm, and BA with a wavelength of 600–660 nm. All images were processed in Adobe Photoshop.

### Estimation of growth and biomass

Batches were inoculated at 10^6^ cells/mL, using freshly growing cells. Growth was estimated by counting cells using a Neubauer cell counting chamber. Cultures were harvested using 1.2-µm Isopore membrane filters (RTTP; Merck Millipore, Cork, IRL). After empty filters were pre-weighed in the exponential (3 days of subculture), transition (exponential to stationary, 5 days of subculture), and stationary phase (7 days of subculture), 10 mL of cultures were filtered. The filters were dried in a 65 °C chamber for 24 h and weighed. Then, the dried cell weight was measured.

### Analysis of total lipid content and fatty acid composition

Dry cell weight for lipid analysis was measured by drying the filtered cells on glass microfiber filters (Whatman, England) at 105 °C for 12 h. The fatty acid methyl esters (FAMEs) were analyzed using an acid-catalyzed transesterification protocol provided by MIDI Inc. (USA) [46]. All analyses were carried out for three biological replicates.

### Measurement of photosynthesis parameters

The O_2_-evolution rate of the microalgae was measured using a Clark-type O_2_ probe (Hansatech Instruments Ltd., Norfolk, UK) as described by Park et al. [47], with some modifications. A dark chamber where 1 mL of cell suspension adjusted to 8 × 10^6^ cells/mL was added and illuminated with an LED lamp. O_2_-evolution was measured at 20, 40, 60, 80, 100, 300, 500, and 1000 μmol photons m^−2^ s^−1^; cells were exposed to each light intensity for 2 min after dark incubation for 10 min. The O_2_-evolution rate was measured and normalized based on the number of cells.

The efficient quantum yield of photosystem II, Y(II) was measured and calculated by a fluorescence imaging system (IMAGING-PAM, Heinz-Walz, Effeltrich, Germany) [48]. Cells in exponential phase were concentrated to 10^7^cells/ml and incubated in the dark for 30 min. After dark adaptation, the fluorescence was measured with a saturating light pulse at intensity 10, and actinic light at intensity 4 was supplied by 450-nm light-emitting diodes (LEDs).

### Southern blot analysis

For Southern blot analysis, each genomic DNA was prepared from WT and transformants. 5 μg of genomic DNA digested with a restriction enzyme (EcoRI) was separated on a 0.8% agarose gel and transferred onto a Hybond-N^+^ membrane (GE Healthcare, USA). *Shble* in PhaT-EF2-PtPTP was amplified by PCR with specific primers (Additional file 1: Fig. S2). According to the manufacturer’s instructions, the probes were labeled by Amersham AlkPhos Direct Labelling Reagents (GE Healthcare), and hybridized probes were detected by Amersham CDP-Star Detection Reagents (GE Healthcare).

## Supplementary information

**Additional file 1.** Additional table and figures.

## Data Availability

All data generated or analyzed during this study are included in this published article and Additional file 1.

## Abbreviations

PTP: Pyruvate transporter-plastid type
BASS2: Bile acid:sodium symporter family protein 2
qRT-PCR: Quantitative real-time PCR
TBP: TATA-box binding protein
FAMEs: Fatty acid methyl esters
GC–MS: Gas chromatography–mass spectrometry
PUFA: Polyunsaturated fatty acids
EF2: Elongation factor 2
PYC2: Pyruvate carboxylase 2
PPDK: Pyruvate–phosphate dikinase
PDC: Pyruvate dehydrogenase complex
OAA: Oxaloacetate
PEP: Phosphoenolpyruvate
