# Supplementary material for: Enhanced pyruvate metabolism in plastids by overexpression of putative plastidial pyruvate transporter in Phaeodactylum tricornutum
Source: Biotechnol Biofuels. 2020 Jul 10;13:120. doi: 10.1186/s13068-020-01760-6 (PMC7350735; doi:10.1186/s13068-020-01760-6)

**Fig S1.** Photosynthesis parameters in WT and overexpression strains

1. Photosynthetic productivity of the WT, PtPTP-OE1, and PtPTP-OE2, measured by O_2_ evolution. (b) Their efficient quantum yield of photosystem Ⅱ, Y(Ⅱ), measured by IMAGING-PAM-M. Error bars, SD of three biological replicates.


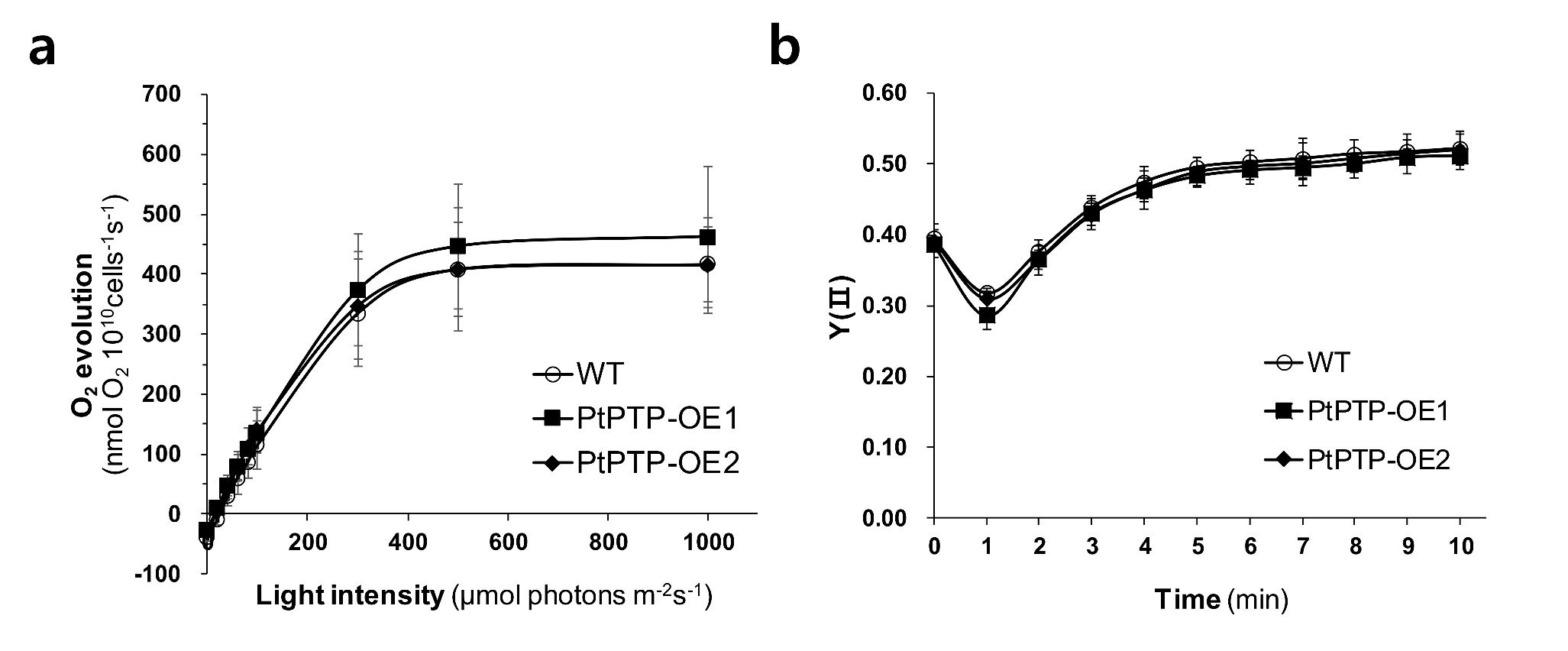


**Table S1.** List of primers used for genomic PCR, and quantitative real-time PCR

|  | **Forward primer** | **Reverse primer** |
| --- | --- | --- |
| **Genomic PCR primers** |  | |
| Primer set for PCR of PTP  (PTP-iFw, PTP-iRv) | 5’-GCAGTCCTGTCGACCACTAC-3’ | 5′-GAAAAGGACGGTCCAGAGGG-3′ |
| PCR of exogenous EF2::PTP  (EF2-iFw, PTP-iRv) | 5′-AAAACCCTACGGGGTGAAAGGAG-3′ | 5′-GAAAAGGACGGTCCAGAGGG-3′ |
| ITS | 5′-TCCGTAGGTGAACCTGCGG-3′ | 5′-TCCTCCGCTTATTGATATGC-3′ |
| **Southern blot analysis** |  | |
| PCR primers for synthesizing probe | 5'-CGACGTGACCCTGTTCATCA-3' | 5'-TTAGTCCTGCTCCTCGGCCA-3' |
| **Quantitative real-time PCR primers** |  | |
| PtPTP (pyruvate transporter-plastid type) | 5’-TGGATGGCCTTGATCGGTTC-3’ | 5’-AACGCTCGCTTGGGACTAAA-3’ |
| Pyruvate dehydrogenase complex  (pyruvate dehydrogenase complex; subunit E1, GenBank accession no. XM_002180298) | 5'-TTGCTCCCGTGAAAATGATG-3' | 5'-GCCATGCAGGTACGTCAAAA-3' |
| PYC2 (pyruvate carboxylase 2, GenBank accession no. XM_002183870) | 5'-TCCAGTCCAAGCAACTCGGT-3' | 5'-TGCCAAGTCACCGACAACCT-3' |
| PPDK (pyruvate, phosphate dikinase, GenBank accession no. XM_002182336) | 5'-AACGTGTCAAGAAAGAGCGTGG-3' | 5'-GCAGAGTCGCTTTCCGTCTT-3' |
|  |  |  |

**Fig S2.** Southern blot analysis of wild type (WT) and PtPTP overexpression lines (PTP-OE1 and -OE2).

Genomic DNA (5µg) was digested with *EcoR*Ⅰ. The partial DNA fragment of *Sh ble* was used as a probe. SM : DNA size marker.


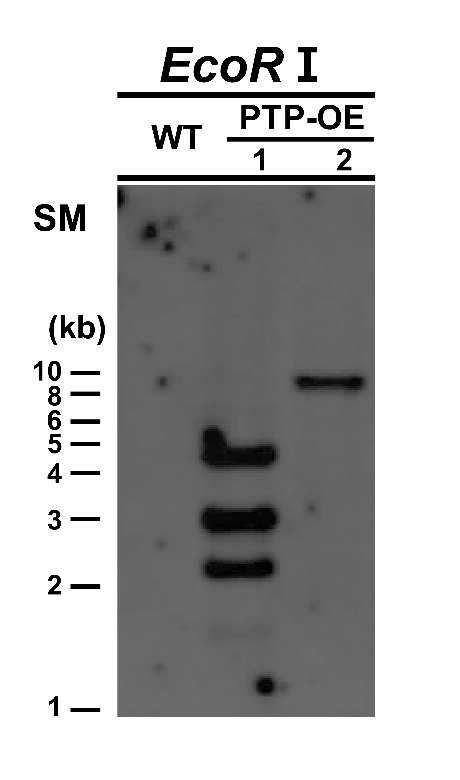

Supplement: Supplementary file 1 — Additional file 1. Additional table and figures. [file 13068_2020_1760_MOESM1_ESM.docx]
